# Oxidative coupling of N-nitrosoanilines with substituted allyl alcohols under rhodium (III) catalysis

**DOI:** 10.3389/fchem.2024.1506493

**Published:** 2024-12-17

**Authors:** Priyanka Chaudhary

**Affiliations:** ^1^ Department of Chemistry, University of Lucknow, Lucknow, India; ^2^ Department of Chemical Engineering, Yeungnam University, Gyeongsan, Republic of Korea

**Keywords:** N-nitrosamines, oxidative coupling, allyl alcohols, rhodium (III) catalysis, C-H activation and functionalization

## Abstract

Rhodium(III) catalysis has been used for C-H activation of *N*-nitrosoanilines with substituted allyl alcohols. This method provides an efficient synthesis of the functional *N*-nitroso *ortho* β-aryl aldehydes and ketones with low catalyst loading, high functional group tolerance, and superior reactivity of allyl alcohols toward *N*-nitrosoanilines. We demonstrated that reaction also proceeds through the one-pot synthesis of *N*-nitrosoaniline, followed by subsequent, C-H activation. The protocol was also feasible with acyrlaldehyde and methyl vinyl ketone which furnished the same oxidative *N*-nitroso coupling product.

## Introduction

Transition metal-catalyzed reactions have a great impact on the synthesis of natural products and in medicinal chemistry (Crawley and Trost, 2011; Du Bois, 2011; Hayler et al., 2019; Brandsma et al., 1999). They have been proven to be the promising and key-driven strategy for the C-H activation of functionalized arenes. In this context, traditional palladium-catalyzed C-H olefination (the oxidative Heck reaction or Fujiwara–Moritani reaction) has been known for generating a new C-C bond (Oestreich, 2009; Moritani and Fujiwara, 1967; Fujiwara et al., 1969). Later, in 2000, Matsumoto disclosed the rhodium-catalyzed oxidative Heck reaction (Matsumoto and Yoshida, 2000; Matsumoto et al., 2002). Furthermore, various research groups have significantly contributed toward rhodium-catalyzed C-H functionalization (Colby et al., 2009; Tian and Loh, 2015; Huang et al., 2013; Li B. et al., 2015; Deng et al., 2016; Kim et al., 2017; Font et al., 2018; Wu et al., 2019; Shi et al., 2020). In this respect, transitory directing groups have reached a remarkable milestone. Indeed, among the various nitrogen-based directing groups (amide, amine, imine, pyridine, pyrimidine, and pyrazole), we focused on *N*-nitrosoanilines with all the key features, which can be transformed to various prevalent structural motifs with significant synthetic and biological importance.

**SCHEME 1:**
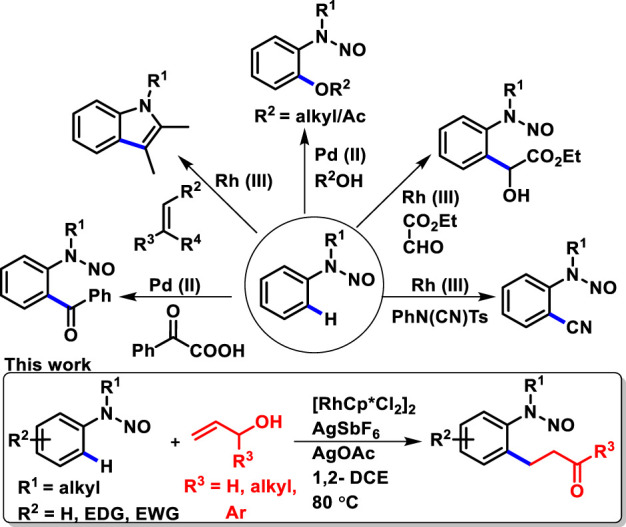
Recent reports of metal-directed C-H activation of *N*-nitrosoanilines and this work.

*N*-nitrosamines are chemical compounds that are extensively found in naturally active molecules and also in a range of food and cosmetic products (Loeppky and Outram, 1982; Loeppky and Michejda, 1994; Wang et al., 2005). The chemistry of *N*-nitrosamines has gained interest in organic and medicinal chemists. In this respect, *N*-nitrosoanilines are found to be a very attractive directing group as they can be easily removed and installed (Chaudhary et al., 2016a; Chaudhary et al., 2018). *N*-nitrosamines belong to a versatile class of compounds as they serve as important building blocks. They have become valuable intermediates, which are generally used as an anchor for further transformation, such as reduction to hydrazines (Hartman and Roll, 1933; Chaudhary et al., 2016b) and amines (Chaudhary et al., 2018), synthesis of mesoionic–heterocyclic compound sydnones (Stewart, 1964; Browne and Harrity, 2010) and aryl C-nitroso compounds through Fischer–Hepp rearrangement (Williams, 1975; Cikotiene et al., 2013), and, also, derivatization at the α-carbon of *N*-nitrosamines (Seebach and Enders, 1975). In addition to these, recently, *N*-nitrosoanilines have emerged as a traceless directing group since the nitroso functionality possesses the lone pair which can coordinate with the transition metal for the activation of the inert C-H bond (Lee et al., 2002). Several research groups have developed metal-catalyzed *ortho*-functionalization of *N*-nitrosoanilines, for example, alkenylation, acylation, alkoxylation, acyloxylation, and cyanation (Scheme 1) (Liu B. et al., 2013; Wu et al., 2016; Gao and Sun, 2014; Li D.-D. et al., 2015; Dong et al., 2015; Huang et al., 2016; Xiong et al., 2023). Despite this recent progress in the C-H functionalization of *N*-nitrosoanilines, to the best of our knowledge, the oxidative alkylation of *N*-nitrosoanilines with allyl alcohols has not been reported. Allyl alcohols serve as immensely important building blocks in organic synthesis and have been explored as a reaction partner for C-H functionalization (Wang et al., 2018; Chen et al., 2012; Wang et al., 2019; Ouyang et al., 2022). Herein, we report rhodium-catalyzed regioselective *ortho* C-H oxidative alkylation of *N*-nitrosoanilines with various substituted allyl alcohols to provide valuable functional *N*-nitroso *ortho* β-aryl aldehydes and ketones (Scheme 1).

Our investigation to optimize reaction conditions began with the reaction of *N*-methyl *N*-nitrosoaniline (**1a**) and allyl alcohol (**2a**) under different catalysts, additives, and solvents (Table 1). Initially, we studied that the reaction of **1a** with **2a** in the presence of [RhCp*Cl_2_]_2_ (5 mol%) and AgSbF_6_ (10 mol%) in 1,2-dichloroethane (DCE) at 80°C for 12 h did not provide any product. (Table 1, entry 1). Therefore, the role of additives was examined for any improvement in the reaction. The employment of 100 mol% of Cu(OAc)_2_, Ag_2_CO_3_, NaOAc, and AgOAc in dichloroethane at 80°C for 12 h afforded **3a** with 60, 46, 31% and 67% yields, respectively (Table 1, entries 2–5). An increase or decrease in the amount of AgOAc from 200 mol% to 50 mol% did not affect the yield of the desired product **3a** (Table 1, entries 6 and 7). However, decline in the loading of the [RhCp*Cl_2_]_2_ catalyst to 2.5 mol% along with AgSbF_6_ (10 mol%) and 100 mol% of AgOAc in DCE at 80°C for 12 h provided a high yield of **3a** (82%) (Table 1, entry 8). The product **3a** was obtained as *syn-* and *anti*-isomers in 1: 0.13 ratio. Furthermore, the decrease in the amount of the [RhCp*Cl_2_]_2_ catalyst to 1 mol% was experimented for 12 h, which lowered the yield of **3a** to 75% (Table 1, entry 9). However, any increase or decrease in the amount of AgSbF_6_ from 20 mol% to 5 mol% did not enhance the yield (Table 1, entries 10 and 11). Later, the effect of the solvents was explored. In non-polar and polar solvents such as CH_3_CN, 1,4-dioxane, THF, and MeOH, **3a** was obtained in 15, 32, 21%, and 11% yields, respectively (Table 1, entries 12–15).

**TABLE 1:** Optimization of reaction conditions^a^.

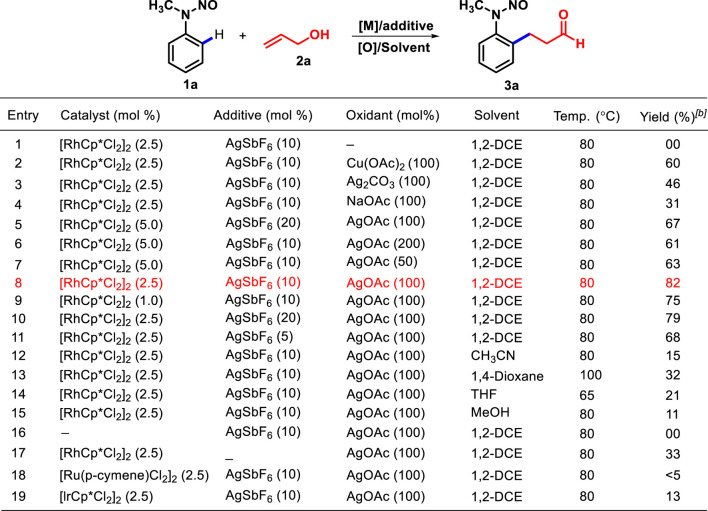

^a^ Reaction conditions: **1a** (0.5 mmol) and **2a** (0.6 mmol) in the solvent (3 mL) under a N_2_ atmosphere.

^b^ Isolated yield after column chromatography.

**FIGURE 1:**
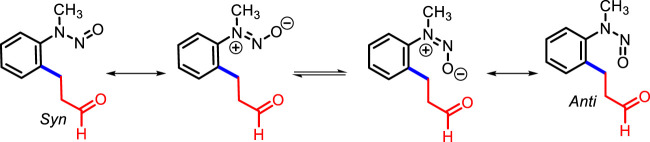
*Syn* and *anti* orientation of **3a** (Liu B. et al., 2013).

In the absence of the [RhCp*Cl_2_]_2_ catalyst, no product was detected with AgSbF_6_ and AgOAc in 1,2-DCE (Table 1, entry 16), whereas the absence of AgSbF_6_ leads to a lower yield of **3a** (33%) (Table 1, entry 17) in the presence of 2.5% [RhCp*Cl_2_]_2_. Furthermore, other catalysts such as [Ru (*p*-cymene)Cl_2_]_2_ and [IrCp*Cl_2_]_2_ were found to be less efficient compared to [RhCp*Cl_2_]_2_ for the transformation (Table 1, entries 18 and 19). The ^1^H NMR spectrum of **3a** showed distinctive signals for adjacent methylene (–CH_2_-CH_2_-) protons to aldehyde (δ 2.76 and δ 2.86 ppm as a triplet, *J* = 7.4 Hz), and the aldehyde singlet proton was observed at δ 9.75 ppm. The ^13^C NMR spectrum of **3a** showed a representative signal for the adjacent methylene carbon to aldehyde, which was observed at 44.7 ppm, and the next methylene carbon to it was observed at 23.8 ppm; the carbonyl group of the aldehyde was observed at 200.5 ppm. **3a** was obtained as a mixture of *syn* and **anti** at a ratio of approximately 1: 0.13 (determined using the ^1^H NMR spectrum) with 82% yield (Figure 1).

Having established the optimized condition, the reaction of substituted *N*-nitroso *N*-alkyl anilines was investigated with allyl alcohol. Treatment of **2a** with several substituted *N*-nitroso *N*-alkyl anilines, namely, **1b**–**1m,** bearing electron-donating and electron-deficient groups, was observed (Table 2). The *p*-substituted electron-donating *N*-nitroso *N*-methyl anilines (methyl and isopropyl) were converted to the corresponding products **3b** and **3c** with good yields (72% and 75%, respectively). Similarly, the substrates bearing halide groups such as Br, Cl, and F at the *para* position under optimized conditions provided the desired products **3d**–**3f** with good yields (60%–73%). It is noteworthy that other *p*-substituted functionalities that have strong electron-withdrawing tendencies, such as trifluoromethyl, cyano, nitro, ester, and acetyl groups, provided the expected products **3g**–**3k** with 62%–77% yields in 12 h.

**TABLE 2:** Substrate scope of various *N*-nitroso *N*-alkyl nitrosoanilines **1b**–**1m** and allyl alcohol **2a** ^a^ ^,^ ^b^ ^,^ ^c^.

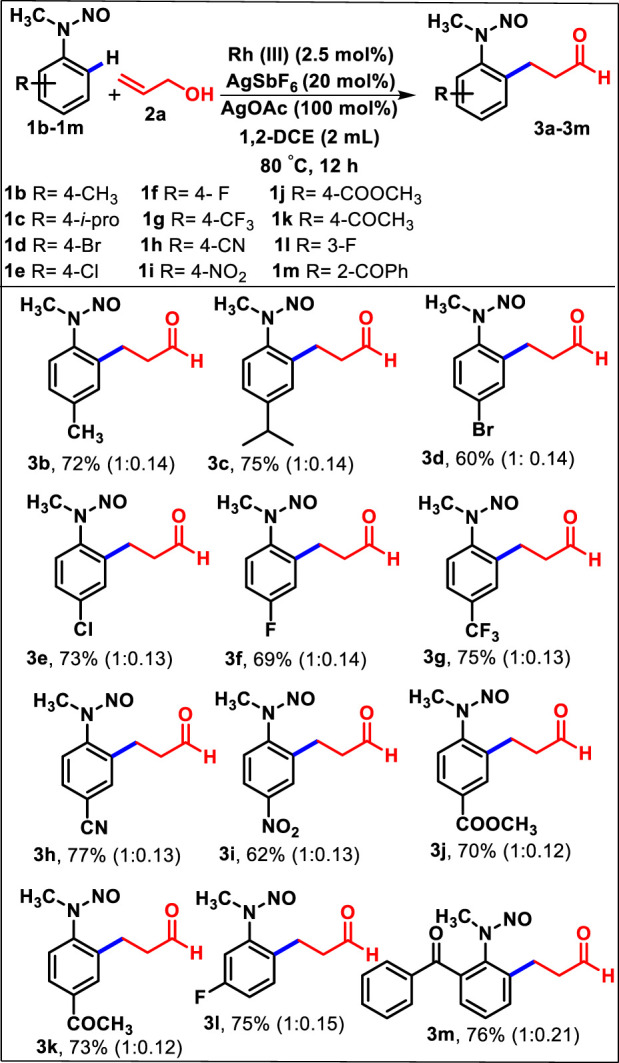

^a^ Reactions were performed with **1** (0.5 mmol, 1.0 equiv.) and **2** (0.6 mmol, 1.2 equiv.) in DCE (3 mL) at 90°C for 12 h under a N_2_ atmosphere.

^b^ Isolated yield.

^c^ Ratio of *syn* to *anti* isomers, determined using the 1H NMR spectrum.

The oxidative alkylation of *m*-fluoro *N*-methyl *N*-nitrosoaniline also yielded the expected product **3l** away from the sterically hindered position with 75% yield. Later, the attempted reaction of sterically hindered **1m** with **2a** regioselectively afforded the *ortho*-substituted (2-benzoyl-6-(3-oxopropyl) phenyl)-*N*-methyl *N*-nitrosoaniline (**3m**) smoothly with 76% yield. Overall, the simple allyl alcohol underwent C-C bond formation effectively with a range of *N*-nitrosoanilines of varying electronic and steric factors.

On account of these results, under the assistance of *N*-nitroso as a directing group, we attempted to explore the reactivity of substituted allyl alcohol **2b**–**2d** toward different *N*-alkyl *N*-nitrosoanilines (Table 3). The unsubstituted nitrosoaniline reacted with 1-methyl and 1-ethyl, which yielded the corresponding products **4a** and **4b** in 72% and 70%, respectively. Similarly, the oxidative alkylation of the *p*-substituted electron-donating substrate (-Me and -^*i*^Pr) provided the corresponding β-aryl ketones **4c** and **4d** in good yields (78% and 79%). In addition, aryl halides such as bromide, chloride, and fluoride at the *p*-position were found to be stable during the reaction conditions and produced the products **4e**, **4f**, and **4g** with 80%, 83%, and 81% yield, respectively. Furthermore, the sensitive functionalities, such as 4-CF_3_, 4-CN, 4-COOCH_3_, and 4-COCH_3_, on the benzene ring of *N*- nitrosoanilines were allowed to react with **2b**, as a result of which **4h**–**4l** were successfully isolated with 73%–80% yield. Moreover, the reaction of **2b** with *m*-substituents such as methyl and fluoro nitrosoanilines afforded the corresponding products **4m** and **4n** with 71% and 70% yield, respectively. C-H activation occurred toward a less sterically hindered position of nitrosoaniline. The variation in *N*-alkyl substitution of 2-methyl *N*-nitrosoaniline from methyl **1o’** to ethyl **1p’** was found to have well-participated under the standard reaction conditions, and products **4o** and **4p** were obtained in good yields (70%–72%). The other *N*-methyl nitrosoanilines containing *o*-substituents such as -Ph and -COPh provided the products **4q** and **4r** in 75% and 74% yields, respectively. This indicates that the protocol has the least influence of steric encumbrance of *ortho*-substituents. Intriguingly, we investigated that the reaction of **1a’** with 1-phenyl allyl alcohol (**2d**) yielded the products **4s** and **4t** in 32% and 17% yields, respectively. Moreover, the combination of **1a’** with pent-3-en-2-ol (**2e**) was also inspected, but no product was observed. This may be due to the steric influence of the methyl group of **2e,** which hindered the oxidative coupling to *N*-methyl *N*-nitrosoaniline (**1a’**).

**TABLE 3:** Substrate scope of various *N*-nitroso *N*-alkyl nitrosoanilines **1a’**-**1u’** and substituted allyl alcohol **2b**-**2e** ^a^ ^,^ ^b^ ^,^ ^c^.

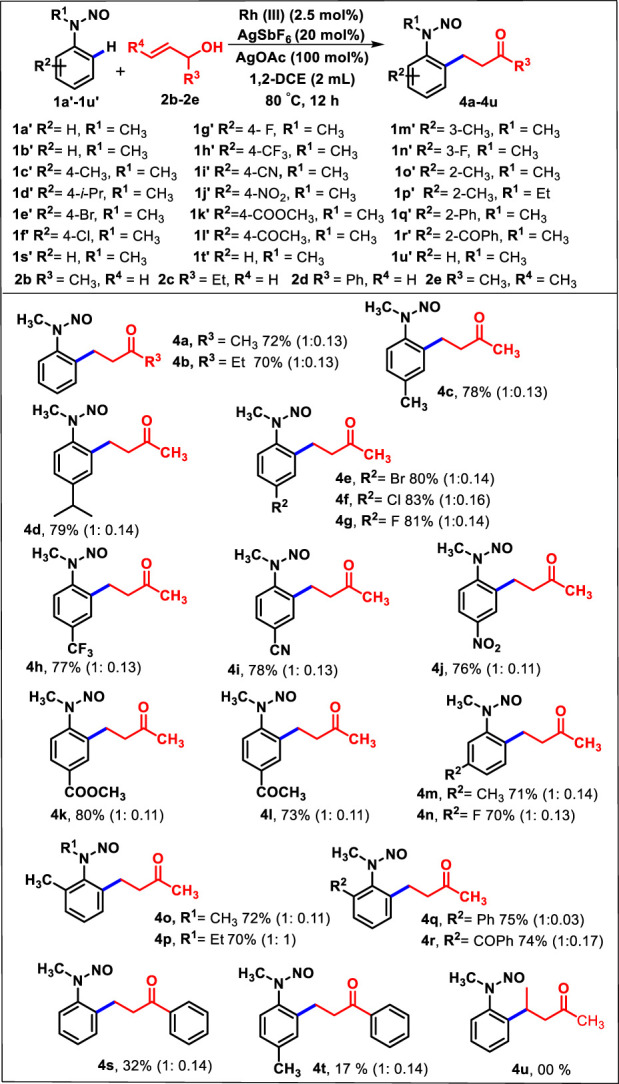

^a^ Reactions were performed with **1** (0.5 mmol, 1.0 equiv.) and **2** (0.6 mmol, 1.2 equiv.) in DCE (3 mL) at 90°C for 12 h under a N_2_ atmosphere.

^b^ Isolated yield.

^c^ Ratio of *syn* to *anti* isomers, determined using the 1H NMR spectrum.

**SCHEME 2:**
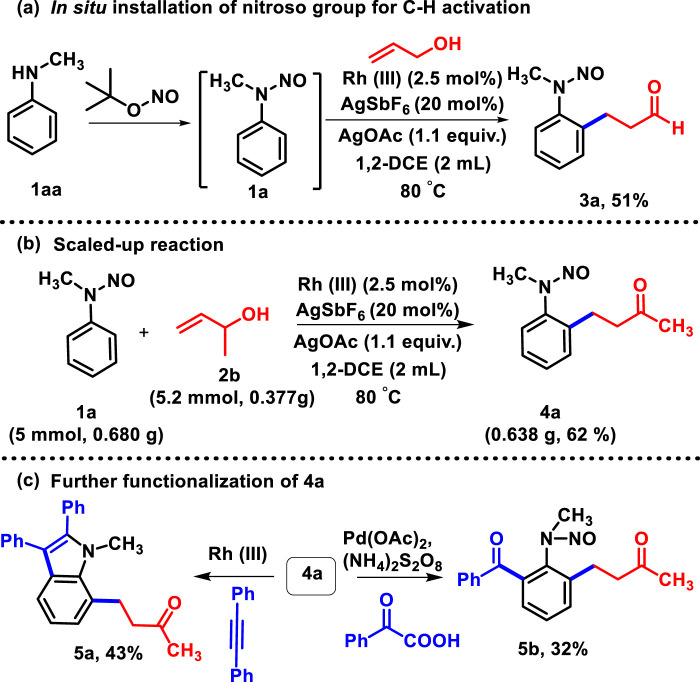
**(A)** *In situ* addition of the *N*-nitroso group to *N*-methyl aniline for C-H activation. **(B)** Scaled-up reaction. **(C)** Further transformation of **4a**.

Regarding C_sp2_-C_sp2_ bond formation, we attempted one-pot synthesis (Hayashi, 2016), where the addition of the nitroso group (Chaudhary et al., 2018) to *N*-methyl aniline (**1aa**) leads to the corresponding nitrosoaniline, and fortunately, we obtained the oxidative coupled product (**3a**) under the standard reaction conditions with 51% yield (Scheme 2A).

**SCHEME 3:**
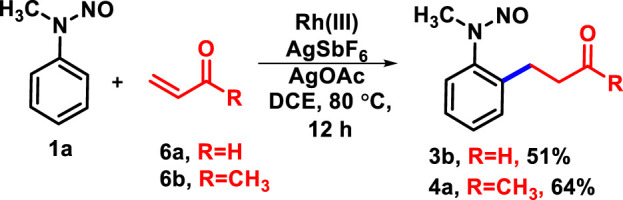
C-H activation of *N*-nitrosoaniline with α,β-unsaturated aldehyde and ketone under standard conditions.

Henceforth, the protocol exemplifies that implementation of a nitroso unit and subsequent oxidative C-H activation with allyl alcohol reduces time and labor, which, obviously, displays its advantages. To show the synthetic utility of the protocol, the gram-scale synthesis of **4a** had been performed (Scheme 2B) and the product was obtained with 62% yield, which was utilized for different transformation processes (Scheme 2C). Versatile *N*-nitroso directing allowed further catalyzation of C-H activation of **4b** at the other *ortho*-position with rhodium (Liu B. Q. et al., 2013) and palladium (Wu et al., 2016). The activation with diphenyl acetylene and phenyl glyoxylic acid yielded the desired products **5a** and **5b** with 43% and 32% yields, respectively. In the course of our study, we performed the reaction of *N*-nitrosoaniline under the same reaction conditions with acrylaldehyde and methyl vinyl ketone. To the best of our knowledge, only β-hydride-eliminated products were obtained such as **3b** and **4a**; no olefinated or protonolysis product was observed (Scheme 3) (Sun et al., 2010; Peng et al., 2012).

**SCHEME 4:**
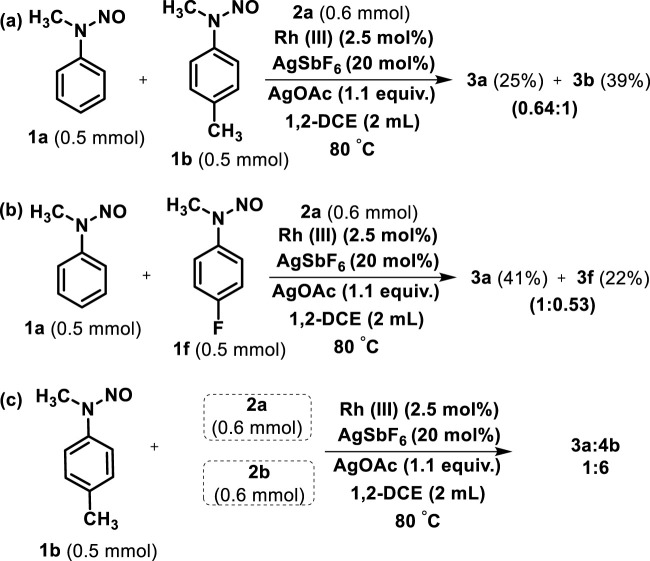
**(A)** Chemoselectivity of between *N*-methyl *N*-nitrosoanilines **1a** and **1b** with allyl alcohol (**2a**). **(B)** Chemoselectivity of between *N*-methyl *N*-nitrosoanilines **1a** and **1f** with allyl alcohol (**2a**). **(C)** Chemoselectivity of 4-methyl *N*-methyl *N*-nitrosoaniline (**1b**) with allyl alcohol (**2a**) and 1-methyl allyl alcohol (**2b**).

The chemoselectivity of two different *N*-methyl *N*-nitrosoanilines toward allyl alcohol (**2a**) was examined under the standard reaction conditions (Scheme 4A). First, the reaction of the unsubstituted (**1a**) and 4-methyl *N*-methyl *N*-nitrosoaniline (**1b**) subjected to react with allyl alcohol (**2a**) for 12 h led to the respective products **3a** and **3b** with 25% and 39% yields, respectively (Scheme 4; Scheme 4A). Similarly, treatment of unsubstituted (**1a**) and 4-fluoro *N*-methyl *N*-nitrosoaniline (**1f**) with **2a** afforded the products **3a** and **3f** with 41% and 22% yields, respectively (Scheme 4B). The results indicated that the *N*-nitrosoaniline bearing the electron-donating group is more chemoselective than the unsubstituted and electron-withdrawing bearing substrate. Even the chemoselectivity of 4-methyl *N*-methyl *N*-nitrosoaniline (**1b**) toward allyl alcohol (**2a**) and 1-methyl allyl alcohol (**2b**) was also investigated, which demonstrated the formation of **3a**:**4b** in the ratio of 1:6 at the same refractive index (*R*_*f*_) (Scheme 4C). These intermolecular competitive reactions indicate the simultaneous formation of rhodium carbon and the cleavage of the C-H bond probably by a concerted metalation mechanism (CMD) (Lapointe and Fagnou, 2010).

**SCHEME 5:**
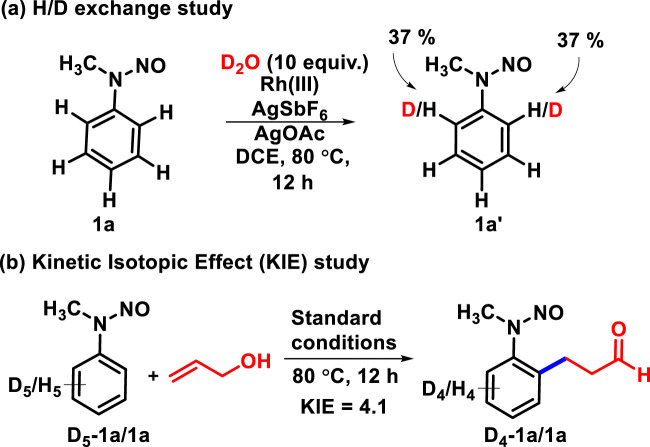
Preliminary mechanistic studies. **(A)** H/D Exchange Study. **(B)** Kinetic Isotopic Effect (KIE) Study.

To gain mechanistic insights, we conducted deuterium labeling experiments, as shown in Scheme 5. The H/D exchange experiment was carried out with 2.5 mol% Rh(III), AgSbF_6_ (10 mol%), and AgOAc (1 equiv.) to yield **1a** (Scheme 5A). The incorporation of 37% deuterium was observed at *ortho* positions of **1a,** which revealed that the C-H activation step is reversible. The parallel kinetic isotopic effect (KIE) was evaluated through an experiment with **D**_**5**_ **-1a** and **1a** and was found to be *K*_*D*_ */K*_*H*_ = 4.1. This interprets that the C-H bond activation step probably is the rate-determining step (Scheme 5B).

**SCHEME 6:**
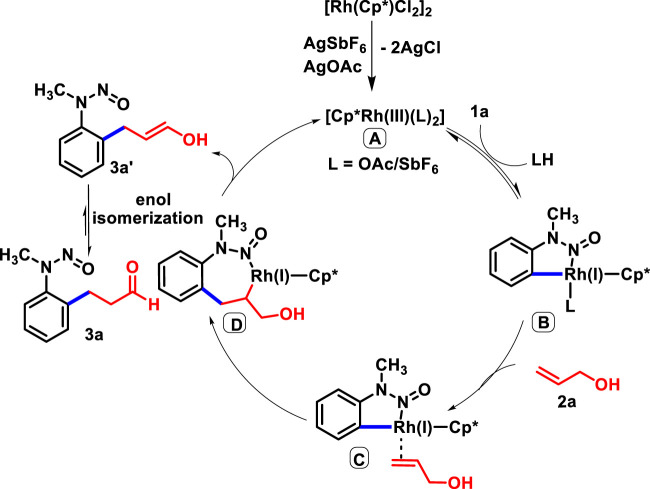
Proposed mechanism.

Based on the deuterium labeling mechanistic studies and available literature, we depicted the catalytic cycle. The foremost step involves the *ortho* C-H activation of **1a** with the active rhodium catalyst **A,** which yields five-membered rodacycle **B**. Subsequently, the co-ordination of **2a** leads to **C**, which undergoes migratory insertion to generate seven-membered rodacycle **D**. The β-hydride elimination of **D** produces **3a’,** which undergoes enol isomerization to yield the desired product **3a** with concomitant regeneration of the Rh(III) catalyst for the next catalytic cycle (Scheme 6).

In conclusion, we have developed efficient Rh(III)-catalyzed C-H functionalization of *N*-nitrosoanilines using substituted allyl alcohols. The protocol was applied to a wide range of substrates which gave good yields of products with high functional group tolerance. The protocol provides rapid access for one-pot C-H activation and, also, feasible C-H activation of *N*-nitrosoanilines with α,β-unsaturated carbonyls. Low catalyst loading, great functional group tolerance, and superior reactivity of *N*-nitrosoanilines with substituted allyl alcohols are some of the key features of this protocol.

## Data Availability

The original contributions presented in the study are included in the article/Supplementary Material; further inquiries can be directed to the corresponding author.
