# Supplementary material for: Oxidative coupling of N-nitrosoanilines with substituted allyl alcohols under rhodium (III) catalysis
Source: Front Chem. 2024 Dec 17;12:1506493. doi: 10.3389/fchem.2024.1506493 (PMC11685039; doi:10.3389/fchem.2024.1506493)
Supplement: Supplementary file 1 [file DataSheet1.docx]

Oxidative Coupling of *N*-Nitrosoanilines with Substituted Allyl Alcohols under Rhodium (III) Catalysis

Priyanka Chaudhary^a^*

^a^Department of Chemistry, University of Lucknow, Lucknow-226007.

^b^ Department of Chemical Engineering, Yeungnam University, Gyeongsan, Republic of Korea.

*Corresponding author: priyankaciitbhu@gmail.com

| **S.No.** | **List of Contents** | | **Page No.** |
| --- | --- | --- | --- |
| 1. | General Information | | 2 |
| 2. | Experimental procedure for C-H functionalized products (**3a-3m** and **4a-4t**) | | 2 |
| 3. | Preliminary mechanistic studies | | 3-4 |
|  | 3.1 | H/D exchange experiment | 3 |
|  | 3.2 | Kinetic Isotope Effect (KIE) Study | 4 |
| 4. | Competitive Reactions | | 5-6 |
| 5 | Additional Experiments | | 7-8 |
|  | 5.1 | *In situ* installation of nitroso group for C-H alkylation | 7 |
|  | 5.2 | Scaled-up synthesis of **4a** | 7 |
|  | 5.3 | Further C-H functionalization of **4a** | 7 |
|  | 5.4 | Experimental procedure for C-H functionalized products from α,β- unsaturated carbonyl (**6a** and **6b**) | 8 |
| 6 | Analytical Data of synthesized compounds | | 9 |
| 7 | References | | 27 |
| 8 | ^1^H NMR and ^13^C NMR spectra of synthesized compounds | | 28 |

1. **General Information**

All experiments were carried out under nitrogen, unless stated otherwise. Solvents and reagents were purchased from Sigma Aldrich and TCI chemicals and used without further purification. Merck precoated silica gel plates (Art. 5554) treated with a fluorescent indicator were used for analytical thin layer chromatography (TLC). Column chromatography was performed using silica gel 9385 (Merck) and ethyl acetate/hexane (1:1) were used as eluents. ^1^H NMR and ^13^C NMR spectra were recorded on VNS (600 and 150 MHz) spectrometer or a Bruker DPX (300 and 75 MHz) spectrometer at the core research support center for natural products and medical materials of Yeungnam University. The NMR spectra recorded in CDCl_3_ using δ = 7.26 and 77.00 ppm as the solvent chemical shifts. All chemical shifts (δ) are expressed in units of ppm and *J* values are given in Hz. Multiplicities are abbreviated as follows: s = singlet, d = doublet, t = triplet, q = quartet, m = multiplet or overlap of nonequivalent resonances, and dd = doublet of doublets. Infrared (IR) spectra were recorded on a PerkinElmer Spectrum Two ^TM^IR spectrometer with frequencies expressed in cm^-1^ and high-resolution mass spectrometry (HRMS) was carried out using a *J*EOL *J*MS-700 spectrometer at the Korea Basic Science Institute. The substituted *N*-nitrosoanilines were prepared by following the reported procedure. *N*-nitrosamines **1a**-**1q** were prepared using literature method.^1^

1. **Experimental procedure for the C-H functionalized products (3a-3m and 4a-4t)**

In an oven dried two-necked reaction vessel, a mixture of *N*-alkyl *N*-nitrosamine **1a** (1 mmol) and allyl alcohol **2a** (1.1 mmol) was dissolved in DCE (3 mL). This was then followed by addition of [RhCp*Cl_2_]_2_ (2.5 mol%), AgSbF_6_ (20 mol%) and AgOAc (2.0 equiv.) under nitrogen atmosphere. The reaction mixture was stirred at 80 ℃, and the reaction was stirred for 12 h. Then, the reaction mixture was cooled to room temperature. The solvent was removed in *vacuo* and the residue was purified by silica gel column chromatography (Hex:EtOAc = 80:20) to obtain the desired products.

1. **Preliminary mechanistic studies**

**3.1 H/D exchange experiment**

**Scheme S1**. Control experiment of H/D exchange.

*N*-methyl *N*-nitrosoaniline (**1a**) (0.2 mmol) was taken in an oven dried two-necked round bottomed flask and dissolved in 3 mL of anhydrous 1,2-dichloroethane (DCE). This was then, followed by the addition of [RhCp*Cl_2_]_2_ (2.5 mol %), AgSbF_6_ (20 mol %), AgOAc (1.1 equiv.), and D_2_O (10 equiv.) under nitrogen atmosphere. The reaction mixture was stirred under reflux condition for 12 h. Then, the solvent was removed *in vacua* and the residue was purified by silica gel column chromatography (Hex:EtOAc = 95: 5) to obtain **1a'**. The two *ortho*-H were deuterated with 37% each.

- 1. **Kinetic Isotope Effect (KIE) Study**

**Scheme S2**. Kinetic isotope effect study.

In an oven dried reaction vessel, a mixture of *N*-methyl *N*-nitrosoaniline (**1a**) (0.2 mmol), and allyl alcohol (**2a**) (0.2 mmol) was dissolved in DCE (2 mL). This was then followed by addition of [RhCp*Cl_2_]_2_ (2.5 mol%), and AgOAc (2.0 equiv.) under nitrogen atmosphere. In another reaction flask, *N*-methyl *N*-nitrosoaniline -2,3,4,5,6-d_5_ (**[D_5_]-1a**) (0.2 mmol) was used instead of **1a**. The two reaction mixtures were stirred under reflux condition for 8 h. The reaction mixture was then cooled to room temperature. The reaction mixtures was combined, the solvent was removed *in vacuo* and the product **[D_4_]-3a/3a** was isolated by silica gel column chromatography (Hex:EtOAc = 80:20) in 23% combined yield (22 mg). The value of *K*_H_/*K*_D_ was calculated based on ^1^H NMR. Here, *K*_H_/*K*_D_ = 3.22/(4.00-3.22) = 4.1.

1. **Competitive Reactions**

In an oven dried two-necked reaction vessel, a mixture of *N*-methyl *N*-nitrosoaniline **1a** (0.5 mmol) or *N*-methyl 4-methyl *N*-nitrosamine **1b** (0.5 mmol) and allyl alcohol **2a** (0.6 mmol) was dissolved in DCE (3 mL). This was followed by addition of [RhCp*Cl_2_]_2_ (2.5 mol%), AgSbF_6_ (20 mol%) and AgOAc (2.0 equiv.) under nitrogen atmosphere. The reaction mixture was stirred at 80 ℃ for 12 h. Then, the reaction mixture was cooled to room temperature. The solvent was removed in *vacuo* and the residue was purified by silica gel column chromatography (Hex:EtOAc = 80:20) to obtain the desired products **3a** (25%, 24 mg) and **3b** (39%, 80 mg).

In an oven dried two-necked reaction vessel, a mixture of *N*-methyl *N*-nitrosoaniline **1a** (0.5 mmol) or *N*-methyl 4-fluoro *N*-nitrosamine **1e** (0.5 mmol) and allyl alcohol **2a** (0.6 mmol) was dissolved in DCE (3 mL). This was followed by addition of [RhCp*Cl_2_]_2_ (2.5 mol%), AgSbF_6_ (20 mol%) and AgOAc (2.0 equiv.) under nitrogen atmosphere. The reaction mixture was stirred at 80 ℃ for 12 h. Then, the reaction mixture was cooled to room temperature. The solvent was removed in *vacuo* and the residue was purified by silica gel column chromatography (Hex:EtOAc = 80:20) to obtain the desired products **3a** (41%, 39 mg) and **3f** (22%, 23 mg).

In an oven dried two-necked reaction vessel, a mixture of *N*-methyl *N*-nitrosoaniline **1a** (0.5 mmol) and allyl alcohol **2a** (0.6 mmol) or 1-methyl allyl alcohol **2b** (0.6 mmol) was dissolved in DCE (3 mL). This was followed by addition of [RhCp*Cl_2_]_2_ (2.5 mol%), AgSbF_6_ (20 mol%) and AgOAc (2.0 equiv.) under nitrogen atmosphere. The reaction mixture was stirred at 80 ℃ for 12 h. Then, the reaction mixture was cooled to room temperature. The solvent was removed in *vacuo* and the residue was purified by silica gel column chromatography (Hex:EtOAc = 80:20) to obtain the desired product (18%, 38 mg).

1. **Additional Experiments**

**5.1 *In situ* installation of nitroso group for C-H activation**

In an oven dried two-necked reaction vessel, *N*-methyl aniline (0.5 mmol) was cooled at 0 ℃ and *tert*-butyl nitrite (0.6 mmol, 1.2 equiv.) (TBN) was added and allowed to stir at room temperature for 5 minutes. Then, followed by addition of allyl alcohol **2a** (1.2 equiv.) and DCE (3mL) with [RhCp*Cl_2_]_2_ (2.5 mol%), AgSbF_6_ (20 mol%) and AgOAc (2.0 equiv.) under nitrogen atmosphere. The reaction mixture was stirred at 80 ℃ for 12 h. Then, the reaction mixture was cooled to room temperature. The solvent was removed in *vacuo* and the residue was purified by silica gel column chromatography (Hex:EtOAc = 80:20) to obtain the desired product **3a** (51%, 49 mg).

**5.2 Scaled-up reaction**

In an oven dried two-necked reaction vessel, a mixture of *N*-methyl *N*-nitrosoaniline **1a** (5 mmol, 680 mg) and 1-methyl allyl alcohol **2a** (5.2 mmol, 377 mg) were dissolved in DCE (3 mL). This was followed by addition of [RhCp*Cl_2_]_2_ (2.5 mol%), AgSbF_6_ (20 mol%) and AgOAc (2.0 equiv.) under nitrogen atmosphere. The reaction mixture was stirred at 80 ℃ for 12 h. Then, the reaction mixture was cooled to room temperature. The solvent was removed in *vacuo* and the residue was purified by silica gel column chromatography (Hex:EtOAc = 80:20) to obtain the desired product **4a** (62%, 638 mg).

**5.3 Further C-H functionalization of 4a**

**(a) Rhodium(III)-Catalyzed Indole Synthesis from 4a**^2^

In an oven dried two-necked reaction vessel, **4a** (0.5 mmol, 103 mg) was added followed by [RhCp*Cl_2_]_2_ (4 mol%), AgSbF_6_ (16 mol%) and NaOAc (0.4 equiv.) under nitrogen atmosphere. Then, the CH_3_CN solution (5 mL) of diphenyl acetylene (0.5 mmol, 133 mg) was added through a syringe. The reaction mixture was stirred at 100 ℃ for 20 h. Then, the reaction mixture was cooled to room temperature. The solvent was removed in *vacuo* and the residue was purified by silica gel column chromatography to obtain the desired product **5a**.

**(b)** **Palladium-Catalyzed acylation of 4a**^3^

In an oven dried two-necked reaction vessel, **4a** (0.5 mmol, 103 mg) and Pd(OAc)_2_ (11.2 mg, 0.05 mmol) was added in diglyme (4 mL) and allowed to stir for 5 minutes. Then, followed by the addition of phenyl glyoxylic acid (1.0 mmol) and (NH_4_)_2_S_2_O_8_ (1.0 mmol). The reaction mixture was stirred at room temperature for 18 h. Then, the reaction mixture was quenched with saturated K_2_CO_3_ and diluted with EtOAc. The organic layer was separated, washed with brine, and dried over Na_2_SO_4_. The organic layer was removed in *vacuo* and purified by silica gel column chromatography to obtain the desired product **5b**.

**5.4 Experimental procedure for C-H functionalized products from α, β-unsaturated carbonyl (6a and 6b)**

In an oven dried two-necked reaction vessel, a mixture of *N*-methyl *N*-nitrosoaniline **1a** (0.5 mmol, 68 mg) and acrylaldehyde **6a** (0.6 mmol, 31 mg) or methyl vinyl ketone **6b** (0.6 mmol, 39 mg) was dissolved in DCE (3 mL). This was followed by addition of [RhCp*Cl_2_]_2_ (2.5 mol%), AgSbF_6_ (20 mol%) and AgOAc (2.0 equiv.) under nitrogen atmosphere. The reaction mixture was stirred at 80 ℃ for 12 h. Then, the reaction mixture was cooled to room temperature. The volatiles was removed in *vacuo* and the residue was purified by silica gel column chromatography (Hex:EtOAc = 80:20) to obtain the desired product **3b** (51%, 49 mg) and **4a** ( 64%, 65 mg).

1. **Analytical Data of synthesized compounds**

| **6.1** *N*-methyl-*N*-(2-(3-oxopropyl)phenyl)nitrous amide (**3a**) | |
| --- | --- |
|  | The title compound was obtained as yellow liquid inseparable mixture of *syn* and *anti* in approximately of 1: 0.13 ratio in 82% yield (79 mg). The residue was purified by column chromatography in silica gel eluting with hexane: EtOAc (80:20), *R_f_* = 0.40; IR (neat) 3412, 2985, 2812, 1602, 1505, 747, 691. ^1^H NMR (600 MHz, CDCl_3_) δ 9.69 (s, 1H × 1), 7.36-7.29 (m, 3H× 1), 7.24 (d, *J* = 7.7 Hz, 2H × 0.40), 7.16 (d, *J* = 7.6 Hz, 1H × 1), 6.87 (d, *J* = 7.7 Hz, 1H × 0.20), 4.03 (s, 3H × 0.40), 3.36 (s, 3H × 1), 2.79 (t, *J* = 7.4 Hz, 2H), 2.69 (t, *J* = 7.4 Hz, 2H). ^13^C NMR (150 MHz, CDCl_3_) δ 200.5, 141.2, 136.6, 130.6, 129.4, 127.9, 127.6, 126.9, 126.6, 125.8, 44.7, 43.6, 35.4, 32.0, 23.8, 23.1. HRMS (ESI) m/z: [M+H]^+^ calcd for C_10_H_13_N_2_O_2_: 193.0972; found: 193.0970. |
| **6.2** *N*-methyl-*N*-(4-methyl-2-(3-oxopropyl)phenyl)nitrous amide (**3b**) | |
|  | The title compound was obtained as yellow liquid inseparable mixture of *syn* and *anti* in approximately of 1: 0.14 ratio in 72% yield (74 mg). The residue was purified by column chromatography in silica gel eluting with hexane: EtOAc (80:20), *R_f_* = 0.33; IR (neat) 3312, 2830, 2695, 1610, 1556, 755, 682. ^1^H NMR (300 MHz, CDCl_3_) δ 9.68 (s, 1H × 1), 7.10 (d, *J* = 7.5 Hz, 2H × 1), 7.04 (d, *J* = 7.0 Hz, 2H × 1.33), 6.75 (d, *J* = 9.3 Hz, 1H × 0.14), 4.01 (s, 3H × 0.42), 3.33 (s, 3H × 1), 2.73 (d, *J* = 5.5 Hz, 2H × 1), 2.67 (m, 2H × 1), 2.32 (s, 3H × 1), 2.28 (s, 3H × 0.42). ^13^C NMR (75 MHz, CDCl_3_) δ 200.7, 139.5, 138.7, 136.3, 131.0, 128.2, 125.6, 44.8, 35.05, 23.6, 21.1. HRMS (ESI) m/z: [M+H]^+^ calcd for C_11_H_15_N_2_O_2_: 207.1128; found: 207.1127. |
| **6.3** *N*-(4-isopropyl-2-(3-oxopropyl)phenyl)-*N*-methylnitrous amide (**3c**) | |
|  | The title compound was obtained as yellow liquid inseparable mixture of *syn* and *anti* in approximately of 1: 0.14 ratio in 75% yield (88 mg). The residue was purified by column chromatography in silica gel eluting with hexane: EtOAc (65:35), *R_f_* = 0.24; IR (neat) 3305, 2922, 2880, 1708, 1677, 789, 559. ^1^H NMR (600 MHz, CDCl_3_) δ 9.73 (s, 1H × 1), 7.20 (t, *J* = 4.0 Hz, 2H × 1), 7.13 (d, *J* = 8.5 Hz, 2H × 1.30), 6.83 (d, *J* = 8.9 Hz, 2H × 0.16), 4.06 (s, 3H × 0.42), 3.39 (s, 3H × 1), 2.93 (m, 1H × 1), 2.82 (t, *J* = 7.3 Hz, 2H × 1), 2.73 (t, *J* = 7.4 Hz, 2H× 1), 1.26 (d, *J* = 6.9 Hz, 6H × 1), 1.22 (d, *J* = 7.0 Hz, 6H × 1). ^13^C NMR (150 MHz, CDCl_3_) δ 200.9, 200.6, 150.43, 138.9, 136.4, 128.6, 127.8, 125.9, 125.7, 125.6, 125.5, 44.9, 44.7, 41.2, 35.5, 33.8, 23.9, 23.8, 23.7, 23.3. HRMS (ESI) m/z: [M+H]^+^ calcd for C_13_H_19_N_2_O_2_: 235.1441; found: 235.1438. |
| **6.4** *N*-(4-bromo-2-(3-oxopropyl)phenyl)-*N*-methylnitrous amide (**3d**) | |
|  | The title compound was obtained as yellow liquid inseparable mixture of *syn* and *anti* in approximately of 1: 0.14 ratio in 60% yield (81 mg). The residue was purified by column chromatography in silica gel eluting with hexane: EtOAc (75:25), *R_f_* = 0.28; IR (neat) 3342, 2961, 2812, 1776, 1650, 770, 698, 490. ^1^H NMR (600 MHz, ) δ 9.68 (s, 1H × 1), 7.47 (d, *J* = 1.9 Hz, 1H × 1), 7.43 (dd, *J* = 8.4, 2.0 Hz, 1H × 1), 7.37 (d, *J* = 10.1 Hz, 1H × 0.27), 7.04 (d, *J* = 8.4 Hz, 1H × 1), 6.75 (d, *J* = 8.0 Hz, 1H × 0.27), 4.03 (s, 3H × 0.43), 3.33 (s, 3H × 1), 2.75 (t, *J* = 7.2 Hz, 2H × 1), 2.70 (t, *J* = 7.2 Hz, 2H × 1). ^13^C NMR (150 MHz, CDCl_3_) δ 200.1, 199.9, 140.2, 138.9, 137.4, 133.5, 132.6, 131.0, 130.7, 127.4, 127.2, 123.8, 123.1, 44.4, 43.3, 35.3, 23.5, 22.8. HRMS (ESI) m/z: [M+H]^+^ calcd for C_10_H_12_BrN_2_O_2_: 271.0077; found: 271.0074. |
| **6.5** *N*-(4-chloro-2-(3-oxopropyl)phenyl)-*N*-methylnitrous amide **(3e)** | |
|  | The title compound was obtained as yellow liquid inseparable mixture of *syn* and *anti* in approximately of 1: 0.13 ratio in 73% yield (82 mg). The residue was purified by column chromatography in silica gel eluting with hexane: EtOAc (80:20), *R_f_* = 0.35; IR (neat) 3442, 2809, 2780, 1725, 1675, 832, 789, 550. ^1^H NMR (600 MHz, CDCl_3_) δ 9.74 (s, 1H × 1), 7.37 (d, *J* = 2.3 Hz, 1H × 1), 7.33 (dd, *J* = 8.4, 2.4 Hz, 1H), 7.28 (d, *J* = 6.9 Hz, 2H× 0.33), 7.16 (d, *J* = 8.4 Hz, 1H × 1), 6.88-6.85 (m, 1H × 0.18), 4.08 (s, 3H × 0.40), 3.39 (s, 3H × 1), 2.81 (t, *J* = 7.0 Hz, 2H × 1), 2.76 (t, *J* = 6.8 Hz, 2H × 1). ^13^C NMR (150 MHz, CDCl_3_) δ 199.8, 144.0, 137.6, 131.4, 131.2, 127.8, 127.8, 127.8, 127.8, 126.0, 124.6, 124.3, 122.5, 44.5, 43.2, 40.8, 35.1, 23.8, 23.0. HRMS (ESI) m/z: [M+H]^+^ calcd for C_10_H_12_ClN_2_O_2_: 227.0582; found: 227.0579. |
| **6.6** *N*-(4-fluoro-2-(3-oxopropyl)phenyl)-*N*-methylnitrous amide **(3f)** | |
|  | The title compound was obtained as yellow liquid inseparable mixture of *syn* and *anti* in approximately of 1: 0.14 ratio in 73% yield (72 mg). The residue was purified by column chromatography in silica gel eluting with hexane: EtOAc (75:15), *R_f_* = 0.31; IR (neat) 3418, 2889, 2697, 1788, 1606, 1105, 845, 955, 574. ^1^H NMR (600 MHz, CDCl_3_) δ 9.69 (s, 1H × 1), 7.15 (m, 1H × 1), 7.01 (m, 2H × 1), 6.95 (d, *J* = 7.4 Hz, 2H × 0.34), 6.85 (m, 1H × 0.18), 4.03 (s, 3H × 0.44), 3.34 (s, 3H × 1), 2.76 (t, *J* = 7.0 Hz, 2H × 1), 2.71 (t, *J* = 7.5 Hz, 2H × 1). ^13^C NMR (150 MHz, CDCl_3_) δ 200.2, 200.0, 162.5 (d, *J*_C-F_ = 247.6 Hz), 139.5 (d, *J*_C-F_ = 7.5 Hz), 139.5 (d, *J*_C-F_ = 3 Hz ), 137.3 (d, *J*_C-F_ = 3 Hz),, 127.8 (d, *J*_C-F_ = 9 Hz), 127.6 (d, *J*_C-F_ = 9 Hz), 117.2 (d, *J*_C-F_ = 22.5 Hz), 116.4 (d, *J*_C-F_ = 22.5), 114.6 (d, *J*_C-F_ = 22.5), 44.3, 43.3, 41.1, 35.6, 29.6, 23.7, 23.1. HRMS (ESI) m/z: [M+H]^+^ calcd for C_10_H_12_FN_2_O_2_: 211.0877; found: 211.0876. |
| **6.7** *N*-methyl-*N*-(2-(3-oxopropyl)-4-(trifluoromethyl)phenyl)nitrous amide (**3g**) | |
|  | The title compound was obtained as pale yellow liquid inseparable mixture of *syn* and *anti* in approximately of 1: 0.13 ratio in 75% yield (81 mg). The residue was purified by column chromatography in silica gel eluting with hexane: EtOAc (80:20), *R_f_* = 0.35; IR (neat) 3329, 2843, 2863, 2916, 1758, 1658, 1012, 1298, 967, 545. ^1^H NMR (600 MHz, CDCl_3_) ^1^H NMR (600 MHz, CDCl_3_) δ 9.70 (s, 1H × 1), 7.60 (s, 1H × 1), 7.57 (d, *J* = 8.2 Hz, 1H × 1), 7.51 (d, *J* = 8.9 Hz, 2H × 0.27), 7.30 (d, *J* = 8.2 Hz, 1H × 1), 7.02 (d, *J* = 8.0 Hz, 1H × 0.13), 4.08 (s, 3H × 0.41), 3.37 (s, 3H × 1), 2.86 (t, *J* = 7.4 Hz, 2H × 1), 2.75 (t, *J* = 7.4 Hz, 2H × 1).^13^C NMR (150 MHz, CDCl_3_) δ 199.8, 143.9, 137.6, 131.2 (d, *J_C-F_* = 32.7 Hz), 127.8 (q, *J_C-F_* = 3.45 Hz), 126.6, 124.0, 124.6 (q, *J_C-F_* = 4.05 Hz), 124.3, 122.5, 44.5, 43.2, 35.1 , 23.8, 23.0. HRMS (ESI) m/z: [M+H]^+^ calcd for C_11_H_12_F_3_N_2_O_2_: 261.0845; found: 261.0843. |
| **6.8** *N*-(4-cyano-2-(3-oxopropyl)phenyl)-*N*-methylnitrous amide (**3h**) | |
|  | The title compound was obtained as pale yellow liquid inseparable mixture of *syn* and *anti* in approximately of 1: 0.13 ratio in 77% yield (83 mg). The residue was purified by column chromatography in silica gel eluting with hexane: EtOAc (80:20), *R_f_* = 0.33; IR (neat) 3321, 2956, 2801, 2250, 1762, 1627, 1692, 957, 586. ^1^H NMR (600 MHz, CDCl_3_) δ 9.69 (s, 1H × 1), 7.66 (s, 1H × 1), 7.61 (d, *J* = 8.2 Hz, 1H× 1), 7.55 (d, *J* = 5.8 Hz, 2H × 0.28), 7.28 (d, *J* = 8.2 Hz, 1H × 1), 7.01 (d, *J* = 8.6 Hz, 0.13H × 1), 4.08 (s, 3H × 0.38), 3.37 (s, 3H × 1), 2.85 (t, *J* = 7.3 Hz, 2H × 1), 2.75 (t, *J* = 7.3 Hz, 2H × 1). ^13^C NMR (150 MHz, CDCl_3_) δ 199.5, 144.7, 138.0, 134.7, 131.4, 131.2, 126.0, 117.7, 113.0, 44.2, 40.6, 34.9, 23.7. HRMS (ESI) m/z: [M+H]^+^ calcd for C_11_H_12_N_3_O_2_: 218.0924; found: 218.0918. |
| **6.9** *N*-methyl-*N*-(4-nitro-2-(3-oxopropyl)phenyl)nitrous amide (**3i**) | |
|  | The title compound was obtained as pale yellow liquid inseparable mixture of *syn* and *anti* in approximately of 1: 0.13 ratio in 62% yield (73 mg). The residue was purified by column chromatography in silica gel eluting with hexane: EtOAc (75:25), *R_f_* = 0.20; IR (neat) 3251, 2952, 2551, 2245, 1892, 1642, 1432, 869, 561. ^1^H NMR (600 MHz, CDCl_3_) δ 9.71 (s, 1H × 1), 8.23 (d, *J* = 2.2 Hz, 1H × 1), 8.16 (d, *J* = 8.6 Hz, 1H × 1), 8.10 (d, *J* = 11.6 Hz, 1H × 0.33), 7.35 (d, *J* = 8.7 Hz, 1H × 1), 7.09 (d, *J* = 8.5 Hz, 1H × 0.11), 4.12 (s, 3H × 0.38), 3.40 (s, 3H × 1), 2.91 (t, *J* = 7.3 Hz, 2H × 1), 2.81 (t, *J* = 7.3 Hz, 2H × 1). ^13^C NMR (150 MHz, CDCl_3_) δ 199.5, 147.4, 146.1, 138.3, 125.9 (d, 2C), 122.7, 44.2, 34.9, 24.0. HRMS (ESI) m/z: [M+H]^+^ calcd for C_10_H_12_N_3_O_4_: 238.0822; found: 238.0821. |
| **6.10** *N*-methyl 4-(methyl(nitroso)amino)-3-(3-oxopropyl)benzoate (**3*j***) | |
|  | The title compound was obtained as pale yellow liquid inseparable mixture of *syn* and *anti* in approximately of 1: 0.12 ratio in 70% yield (88 mg). The residue was purified by column chromatography in silica gel eluting with hexane: EtOAc (70:30), *R_f_* = 0.22; IR (neat) 3386, 2984, 2862, 2650, 1890, 1758, 1692, 1465, 945, 594. ^1^H NMR (600 MHz, CDCl_3_) δ 9.70 (s, 1H × 1), 8.01 (s, 1H × 1), 7.96 (d, *J* = 10.1 Hz, 1H × 1), 7.90 (d, *J* = 8.2 Hz, 1H × 0.30), 7.24 (d, *J* = 8.2 Hz, 1H × 1), 4.07 (s, 1H × 0.14), 3.89 (s, 3H × 0.35), 3.38 (s, 3H × 1), 2.85 (t, *J* = 7.5 Hz, 2H × 1), 2.74 (t, *J* = 7.5 Hz, 2H × 1). ^13^C NMR (150 MHz, CDCl_3_) δ 200.1, 165.9, 144.7, 136.6, 132.0, 130.7, 128.8, 125.3, 52.4, 44.5, 35.1, 23.8. HRMS (ESI) m/z: [M+H]^+^ calcd for C_12_H_15_N_2_O_4_: 251.1026; found: 251.1024. |
| **6.11** *N*-(4-acetyl-2-(3-oxopropyl)phenyl)-*N*-methylnitrous amide (**3k**) | |
|  | The title compound was obtained as pale yellow liquid inseparable mixture of *syn* and *anti* in approximately of 1: 0.12 ratio in 73% yield (85 mg). The residue was purified by column chromatography in silica gel eluting with hexane: EtOAc (75:25), *R_f_* = 0.28; IR (neat) 3413, 2958, 2884, 2250, 1856, 1723, 1622, 1670, 989, 624. ^1^H NMR (600 MHz, CDCl_3_) δ 9.70 (s, 1H × 1), 7.93 (s, 1H × 1), 7.87 (d, *J* = 8.2 Hz, 1H × 1), 7.84 (s, 1H × 0.13), 7.81 (d, *J* = 8.1 Hz, 1H × 0.17), 7.26 (d, *J* = 8.2 Hz, 1H × 1), 6.99 (d, *J* = 8.1 Hz, 1H × 0.12), 4.08 (s, 1H × 0.36), 3.38 (s, 3H × 1), 2.87 (t, *J* = 7.4 Hz, 2H × 1), 2.76 (t, *J* = 7.4 Hz, 2H × 1), 2.58 (s, 3H × 1), 2.54 (s, 3H × 0.36). ^13^C NMR (150 MHz, CDCl_3_) δ 200.1, 196.8, 144.8, 137.3, 136.9, 130.8, 127.6, 125.5, 44.6, 35.1, 26.7, 23.9. HRMS (ESI) m/z: [M+H]^+^ calcd for C_12_H_15_N_2_O_3_: 235.1077; found: 235.1073. |
| **6.12** *N*-(3-fluoro-2-(3-oxopropyl)phenyl)-*N*-methylnitrous amide **(3l)** | |
|  | The title compound was obtained as pale yellow liquid inseparable mixture of *syn* and *anti* in approximately of 1: 0.15 ratio in 75% yield (79 mg). The residue was purified by column chromatography in silica gel eluting with hexane: EtOAc (75:25), *R_f_* = 0.31; IR (neat) 3359, 3025, 2823, 2652, 1789, 1702, 1625, 1012, 1002, 579. ^1^H NMR (600 MHz, CDCl_3_) δ 9.68 (s, 1H × 1), 7.29 (m, 1H × 1), 7.24 (t, *J* = 7.3 Hz, 1H × 0.18), 7.10 (t, *J* = 8.9 Hz, 1H × 1), 7.05 (t, *J* = 8.8 Hz, 1H × 0.25), 6.99 (d, *J* = 8.0 Hz, 1H × 1), 6.70 (d, *J* = 7.8 Hz, 1H × 0.14), 4.04 (s, 3H × 0.44), 3.36 (s, 3H × 1), 2.79 (t, *J* = 7.7 Hz, 2H × 1), 2.69 (t, *J* = 7.7 Hz, 2H × 1). ^13^C NMR (150 MHz, CDCl_3_) δ 200.3, 161.8 (d, *J* = 242 Hz), 142.5 (d, *J_C-F_* = 7.5 Hz), 128.3 (d, *J_C-F_* = 10.5 Hz), 124.9 (d, *J_C-F_* = 16.5 Hz) 121.1 (d, *J_C-F_* = 4.5 Hz), 116.1 (d, *J_C-F_* = 22.5 Hz), 43.7, 35.4, 17.9. HRMS (ESI) m/z: [M+H]^+^ calcd for C_10_H_12_FN_2_O_2_: 211.0877; found: 211.0876. |
| **6.13** *N*-(2-benzoyl-6-(3-oxopropyl)phenyl)-*N*-methylnitrous amide **(3m)** | |
|  | The title compound was obtained as pale yellow liquid inseparable mixture of *syn* and *anti* in approximately of 1: 0.21 ratio in 76% yield (112 mg). The residue was purified by column chromatography in silica gel eluting with hexane: EtOAc (75:25), *R_f_* = 0.30; IR (neat) 3467, 3314, 2810, 1823, 1775, 1654, 1350, 986, 692. ^1^H NMR (600 MHz, CDCl_3_) δ 9.76 (s, 1H × 1), 7.73 (d, *J* = 8.3 Hz, 2H × 1), 7.56 (dt, *J* = 7.3, 3.6 Hz, 2H × 1), 7.51 (t, *J* = 7.6 Hz, 1H × 1), 7.41 (t, *J* = 7.5 Hz, 3H × 1), 4.12 (s, 1H × 0.63), 3.26 (s, 3H × 1), 2.85 (t, *J* = 6.3 Hz, 2H × 1), 2.78 (d, *J* = 7.4 Hz, 2H × 1). ^13^C NMR (150 MHz, CDCl_3_) δ 199.9, 195.4, 139.2, 138.8, 138.2, 136.5, 133.7, 132.4, 130.1, 129.2, 128.4, 127.9, 44.3, 37.0, 23.5. HRMS (ESI) m/z: [M+H]^+^ calcd for C_17_H_17_N_2_O_3_: 297.1234; found: 297.1229. |
| **6.14** *N*-methyl-*N*-(2-(3-oxobutyl)phenyl)nitrous amide **(4a)** | |
|  | The title compound was obtained as pale yellow liquid inseparable mixture of *syn* and *anti* in approximately of 1: 0.13 ratio in 72% yield (74 mg). The residue was purified by column chromatography in silica gel eluting with hexane: EtOAc (80:20), *R_f_* = 0.38; IR (neat) 3327, 3014, 2810, 1721, 1623, 1402, 990, 821, 590. ^1^H NMR (600 MHz, CDCl_3_) δ 7.35-7.25 (m, 3H × 1), 7.23 (m, 1H × 0.27), 7.15 (d, *J* = 7.7 Hz, 1H × 1), 6.85 (dd, *J* = 8.2, 1.4 Hz, 1H × 0.12), 4.02 (s, 1H × 0.38), 3.35 (s, 3H × 1), 2.71 (t, *J*=7.4, 2H × 1), 2.64 (t, *J*=7.4, 2H × 1), 2.03 (s, 3H × 1). ^13^C NMR (150 MHz, CDCl_3_) δ 207.3, 207.1, 141.1, 138.2, 138.4, 137.2, 130.5, 129.8, 129.6, 129.3, 127.6, 127.3, 125.8 (2C), 44.3, 43.2, 42.1, 41.1, 35.4, 29.8, 25.2, 24.6. HRMS (ESI) m/z: [M+H]^+^: calcd for C_12_H_15_N_2_O_2_: 207.1128; found: 207.1124. |
| **6.15** *N*-methyl-*N*-(2-(3-oxopentyl)phenyl)nitrous amide **(4b)** | |
|  | The title compound was obtained as pale yellow liquid inseparable mixture of *syn* and *anti* in approximately of 1: 0.13 ratio in 70% yield (74 mg). The residue was purified by column chromatography in silica gel eluting with hexane: EtOAc (80:20), *R_f_* = 0.38; IR (neat) 3329, 3106, 2799, 1769, 1697, 1368, 990, 852, 561. ^1^H NMR (600 MHz, CDCl_3_) δ 7.35-7.26 (m, 3H × 1), 7.25-7.21 (m, 1H × 0.13), 7.15 (d, *J* = 7.8 Hz, 1H × 1), 6.85 (d, *J* = 7.0 Hz, 1H × 0.12), 4.03 (s, 1H × 0.38), 3.35 (s, 3H × 1), 2.73 (t, *J* = 7.6 Hz, 2H × 1), 2.61 (t, *J* = 7.6 Hz, 2H × 1), 2.30 (q, *J* = 7.3 Hz, 2H × 1), 0.95 (t, *J* = 7.3 Hz, 3H × 1). ^13^C NMR (150 MHz, CDCl_3_) δ 202.2, 209.9, 141.1, 140.9, 138.5, 138.4, 137.4, 130.6, 129.7, 129.8, 129.4, 127.6, 127.4, 125.9 (2C, overlapped), 43.0, 41.9, 35.9, 35.5, 25.4, 24.7, 7.7, 7.6. HRMS (ESI) m/z: [M+H]^+^ calcd for C_12_H_17_N_2_O_2_: 221.1285; found: 221.1282. |
| **6.16** *N*-methyl-*N*-(4-methyl-2-(3-oxobutyl)phenyl)nitrous amide (**4c**) | |
|  | The title compound was obtained as pale yellow liquid inseparable mixture of *syn* and *anti* in approximately of 1: 0.13 ratio in 78% yield (88 mg). The residue was purified by column chromatography in silica gel eluting with hexane: EtOAc (80:20), *R_f_* = 0.37; IR (neat) 3331, 3121, 2926, 1758, 1645, 1375, 894, 798, 509. ^1^H NMR (600 MHz, CDCl_3_) δ 7.12-7.05 (m, 2H × 1), 7.03 (d, *J* = 7.9 Hz, 1H × 1), 6.73 (d, *J* = 8.5 Hz, 1H × 0.13), 4.00 (s, 3H × 0.38), 3.32 (s, 3H × 1), 2.66 (d, *J* = 6.7 Hz, 2H × 1), 2.63 (d, *J* = 7.0 Hz, 2H × 1), 2.32 (s, 3H × 1), 2.03 (s, 3H × 1). ^13^C NMR (150 MHz, CDCl_3_) δ 207.4, 207.2, 139.9, 139.5, 138.7, 137.8, 136.9, 135.8, 131.1, 130.3, 128.4, 128.0, 125.7, 125.5, 44.5, 43.3, 41.2, 35.5, 29.8, 25.2, 24.6, 21.1 (2C, overlapped). HRMS (ESI) m/z: [M+H]^+^: calcd for C_12_H_18_N_2_O_2_: 222.1363; found: 222.1360. |
| **6.17** *N*-(4-isopropyl-2-(3-oxobutyl)phenyl)-*N*-methylnitrous amide (**4d**) | |
|  | The title compound was obtained as pale yellow liquid inseparable mixture of *syn* and *anti* in approximately of 1: 0.14 ratio in 79% yield (88 mg). The residue was purified by column chromatography in silica gel eluting with hexane: EtOAc (80:20), *R_f_* = 0.37; IR (neat) 3259, 3122, 2823, 1768, 1652, 1452, 1004, 896, 541. ^1^H NMR (600 MHz, CDCl_3_) δ 7.18 (d, *J* = 6.4 Hz, 2H × 1), 7.12 (d, *J* = 8.5 Hz, 1H × 1), 6.81 (d, *J* = 7.9 Hz, 1H × 0.18), 4.05 (s, 1H × 0.41), 3.38 (d, *J* = 1.9 Hz, 3H × 1), 2.92 (m, 1H × 1), 2.74 (t, *J* = 7.1 Hz, 2H × 1), 2.68 (t, *J* = 7.4 Hz, 2H × 1), 2.08 (s, 3H × 1), 1.25 (d, *J* = 6.9 Hz, 6H × 1), 1.22 (d, *J* = 6.9 Hz, 6H × 1). ^13^C NMR (150 MHz, CDCl_3_) δ 207.2, 205.8, 150.6, 150.3, 138.9, 137.8, 137.0, 136.0, 128.6, 127.8, 125.8, 125.7, 125.6, 125.3, 44.6, 43.4, 41.2, 35.5, 33.8, 29.8, 25.5, 24.9, 23.8, 23.7. HRMS (ESI) m/z: [M+H]^+^ calcd for C_14_H_21_N_2_O_2_: 249.1598; found: 249.1592. |
| **6.18** *N*-(4-bromo-2-(3-oxobutyl)phenyl)-*N*-methylnitrous amide **(4e)** | |
|  | The title compound was obtained as pale yellow liquid inseparable mixture of *syn* and *anti* in approximately of 1: 0.14 ratio in 80% yield (113 mg). The residue was purified by column chromatography in silica gel eluting with hexane: EtOAc (80:20), *R_f_* = 0.31; IR (neat) 3352, 2985, 2843, 1752, 1641, 1323, 689, 857, 561. ^1^H NMR (600 MHz, CDCl_3_) δ 7.46 (s, 1H × 1), 7.41 (d, *J* = 8.4 Hz, 1H × 1), 7.39-7.34 (m, 2H × 0.31), 7.03 (d, *J* = 8.4 Hz, 1H × 1), 6.73 (d, *J* = 8.2 Hz, 1H × 0.14), 4.02 (s, 3H × 0.43), 3.33 (s, 3H × 1), 2.71-2.67 (m, 2H × 1), 2.67-2.62 (m, 2H × 1), 2.05 (s, 3H × 1). ^13^C NMR (150 MHz, CDCl_3_) δ 206.8, 206.6, 140.7, 140.2, 139.5, 137.5, 137.1, 133.6, 132.7, 130.9, 130.5, 127.5, 127.4, 123.1, 35.4, 29.8, 25.1. HRMS (ESI) m/z: [M+H]^+^ calcd for C_11_H_14_BrN_2_O_2_: 285.0233; found: 285.0228. |
| **6.19** *N*-(4-chloro-2-(3-oxobutyl)phenyl)-*N*-methylnitrous amide (**4f**) | |
|  | The title compound was obtained as pale yellow liquid inseparable mixture of *syn* and *anti* in approximately of 1: 0.16 ratio in 83% yield (88 mg). The residue was purified by column chromatography in silica gel eluting with hexane: EtOAc (80:20), *R_f_* = 0.34; IR (neat) 3312, 2983, 2799, 1712, 1678, 1402, 864, 541. ^1^H NMR (600 MHz, CDCl_3_) δ 7.35 (t, *J* = 2.0 Hz, 1H × 1), 7.30 (m, 1H × 1), 7.26-7.24 (m, 1H × 0.37), 7.23 (t, *J* = 2.1 Hz, 1H × 0.11), 7.14 (d, *J* = 8.4 Hz, 1H × 1), 6.84 (d, *J* = 8.3 Hz, 1H × 0.18), 4.06 (s, 1H × 0.47), 3.37 (s, 3H × 1), 2.75-2.71 (m, 2H × 1), 2.71-2.68 (m, 2H × 1), 2.09 (s, 3H × 1). ^13^C NMR (150 MHz, CDCl_3_) δ 206.5, 139.7, 139.2, 134.9, 130.5, 129.7, 127.8, 127.5, 127.1, 43.9, 42.8, 40.9, 35.3, 29.8, 25.1, 24.4. HRMS (ESI) m/z: [M+H]^+^ calcd for C_11_H_14_ClN_2_O_2_: 241.0738; found: 241.0727. |
| **6.20** *N*-(4-fluoro-2-(3-oxobutyl)phenyl)-*N*-methylnitrous amide (**4g**) | |
|  | The title compound was obtained as pale yellow liquid inseparable mixture of *syn* and *anti* in approximately of 1: 0.14 ratio in 81% yield (91 mg). The residue was purified by column chromatography in silica gel eluting with hexane: EtOAc (80:20), *R_f_* = 0.32; IR (neat) 3341, 3201, 2810, 1781, 1654, 1457, 1101, 878, 519. ^1^H NMR (300 MHz, CDCl_3_) δ 7.18 (m, 1H × 1), 7.03 (m, 2H), 4.06 (s, 3H × 0.43), 3.37 (s, 3H × 1), 2.71 (s, 4H × 1), 2.09 (s, 3H × 1). ^13^C NMR (75 MHz, CDCl_3_) δ 206.6, 162.5 (d, 247.5 Hz), 140.1 (d. *J*_C-F_ = 8.25 Hz), 137.3 (d, *J*_C-F_ = 3 Hz) 127.9 (d, *J*_C-F_ = 9 Hz), 117.2 (d, *J*_C-F_ = 22.5), 114.3 (d, *J*_C-F_ = 22.5 Hz), 43.9, 35.6, 29.8, 25.1. HRMS (ESI) m/z: [M+H]^+^ calcd for C1_1_H_14_FN_2_O_2_: 225.1034; found: 225.1036. |
| **6.21** *N*-methyl-*N*-(2-(3-oxobutyl)-4-(trifluoromethyl)phenyl)nitrous amide **(4h)** | |
|  | The title compound was obtained as pale yellow liquid inseparable mixture of *syn* and *anti* in approximately of 1: 0.13 ratio in 77% yield (105 mg). The residue was purified by column chromatography in silica gel eluting with hexane: EtOAc (80:20), *R_f_* = 0.33; IR (neat) 3402, 3213, 2809, 1765, 1655, 1410, 1204, 1012, 523. ^1^H NMR (600 MHz, CDCl_3_) δ 7.59 (s, 1H × 1), 7.55 (d, *J* = 8.2 Hz, 1H × 1), 7.49 (d, *J* = 5.1 Hz, 1H × 0.26), 7.29 (d, *J* = 8.1 Hz, 1H × 1), 7.00 (d, *J* = 8.7 Hz, 1H × 0.13), 4.06 (s, 3H × 0.39), 3.36 (s, 3H × 1), 2.79 (t, *J* = 7.5 Hz, 2H × 1), 2.69 (t, *J* = 7.5 Hz, 2H × 1), 2.05 (s, 3H × 1). ^13^C NMR (150 MHz, CDCl_3_) δ 206.6, 206.4, 141.7, 143.9, 139.0 (d, *J* = 237 Hz), 131.2 (q, *J*_C-F_ = 33 Hz), 130.9, 128.4, 127.8 (q, *J*_C-F_ = 3 Hz), 127.5, 126.9, 126.6 (2C, overlapped), 126.5, 126.2, 126.1, 124.7 (2C, overlapped), 124.6, 124.4 (q, *J*_C-F_ = 124.4), 122.6, 120.7, 44.0, 42.7, 40.7, 35.1, 29.8 (2C, overlapped), 25.3, 24.5. HRMS (ESI) m/z: [M+H]^+^ calcd for C_12_H_14_F_3_N_2_O_2_: 275.1002; found: 275.1001. |
| **6.22** *N*-(4-cyano-2-(3-oxobutyl)phenyl)-*N*-methylnitrous amide (**4i**) | |
|  | The title compound was obtained as pale yellow liquid inseparable mixture of *syn* and *anti* in approximately of 1: 0.13 ratio in 78% yield (90 mg). The residue was purified by column chromatography in silica gel eluting with hexane: EtOAc (80:20), *R_f_* = 0.31; IR (neat) 3329, 3106, 2799, 1769, 1697, 1368, 990, 852, 561. ^1^H NMR (600 MHz, CDCl_3_) δ 7.65 (s, 1H × 1), 7.59 (dd, *J* = 8.2, 1.6 Hz, 1H × 1), 7.54 (s, 1H × 0.25), 7.27 (d, *J* = 8.2 Hz, 1H × 1), 6.99 (d, *J* = 7.9 Hz, 1H × 0.13), 4.07 (s, 3H × 0.40), 3.36 (s, 3H × 1), 2.78 (t, *J* = 7.2 Hz, 2H × 1), 2.69 (t, *J* = 7.3 Hz, 2H × 1), 2.06 (s, 3H × 1). ^13^C NMR (150 MHz, CDCl_3_) δ 206.2, 144.7, 138.7, 134.7, 133.3, 131.2, 131.0, 126.1, 117.8, 113.0, 43.8, 42.4, 40.6, 35.0, 29.8 (2C, overlapped), 25.1, 24.2. HRMS (ESI) m/z: [M+H]^+^ calcd for C_12_H_14_N_3_O_2_: 232.1081; found: 232.1079. |
| **6.23** *N*-methyl-*N*-(4-nitro-2-(3-oxobutyl)phenyl)nitrous amide **(4*J*)** | |
|  | The title compound was obtained as pale yellow liquid inseparable mixture of *syn* and *anti* in approximately of 1: 0.11 ratio in 76% yield (95 mg). The residue was purified by column chromatography in silica gel eluting with hexane: EtOAc (80:20), *R_f_* = 0.35; IR (neat) 3342, 3197, 2821, 1772, 1689, 1410, 952, 897, 527. ^1^H NMR (600 MHz, CDCl_3_) δ 8.21 (d, *J* = 2.3 Hz, 1H × 1), 8.13 (dd, *J* = 8.6, 2.4 Hz, 1H × 1), 8.09 (s, 1H × 0.11), 8.07 (d, *J* = 6.3 Hz, 1H × 0.11), 7.35 (d, *J* = 8.7 Hz, 1H × 1), 7.08 (d, *J* = 8.5 Hz, 1H × 0.13), 4.11 (s, 3H × 0.34), 3.39 (s, 3H × 1), 2.84 (t, *J* = 7.2 Hz, 2H × 1), 2.76 (t, *J* = 7.2 Hz, 2H × 1), 2.08 (s, 3H × 1). ^13^C NMR (150 MHz, CDCl_3_) δ 206.3, 206.2, 148.2, 147.4, 146.1, 144.9, 141. 1, 138.9, 127.1, 126.1, 125.8, 124.3, 122.6, 122.4, 43.6, 42.3, 40.5, 34.9, 29.8, 29.7, 25.3, 24.4. HRMS (ESI) m/z: [M+H]^+^ calcd for C_11_H_14_N_3_O_4_: 252.0979; found: 252.0976. |
| **6.24** methyl 4-(methyl(nitroso)amino)-3-(3-oxobutyl)benzoate (**4k**) | |
|  | The title compound was obtained as pale yellow liquid inseparable mixture of *syn* and *anti* in approximately of 1: 0.11 ratio in 80% yield (106 mg). The residue was purified by column chromatography in silica gel eluting with hexane: EtOAc (80:20), *R_f_* = 0.37; IR (neat) 3314, 3125, 2817, 1791, 1655, 1451, 987, 854, 585. ^1^H NMR (600 MHz,CDCl_3_) δ 8.05 (s, 1H × 1), 7.99 (d, *J* = 8.2 Hz, 1H × 1), 7.97-7.92 (m, 2H × 0.36), 7.28 (d, *J* = 8.2 Hz, 1H × 1), 6.99 (d, *J* = 8.3 Hz, 1H × 0.18), 4.11 (s, 3H × 0.38), 3.94 (s, 3H × 1), 3.42 (s, 3H × 1), 2.83 (t, *J* = 7.4 Hz, 2H × 1), 2.75 (t, *J* = 7.4 Hz, 2H × 1), 2.11 (s, 3H × 1). ^13^C NMR (150 MHz, CDCl_3_) δ 206.6, 166.0, 144.7, 137.3, 132.0, 130.7, 128.6, 125.5, 52.4, 44.2, 35.1, 29.8, 25.3. HRMS (ESI) m/z: [M+H]^+^ :calcd for C_13_H_17_N_2_O_4_: 265.1183; found: 265.1186. |
| **6.25** *N*-(4-acetyl-2-(3-oxobutyl)phenyl)-*N*-methylnitrous amide **(4l)** | |
|  | The title compound was obtained as pale yellow liquid inseparable mixture of *syn* and *anti* in approximately of 1: 0.11 ratio in 73% yield (91 mg). The residue was purified by column chromatography in silica gel eluting with hexane: EtOAc (80:20), IR (neat) 3323, 3099, 2845, 1718, 1654, 1365, 1023, 898, 556. *R_f_* = 0.35; ^1^H NMR (600 MHz, CDCl_3_) δ 7.93 (s, 1H × 1), 7.86 (d, *J* = 8.2 Hz, 1H × 1), 7.83 (s, 1H × 0.14), 7.79 (d, *J* = 9.8 Hz, 1H × 0.13), 7.26 (d, *J* = 8.2 Hz, 1H × 1), 6.97 (d, *J* = 8.1 Hz, 1H × 0.12), 4.07 (s, 3H × 0.36), 3.37 (s, 3H × 1), 2.79 (t, *J* = 7.4 Hz, 2H × 1), 2.71 (d, *J* = 7.5 Hz, 2H × 1), 2.58 (s, 3H × 1), 2.54 (s, 3H × 0.36), 2.06 (s, 3H × 1). 13C NMR (151 MHz, CDCl_3_) δ 206.9, 206.7, 196.9, 144.8, 142.7, 139.2, 138.0, 137.5, 137.2, 130.7, 129.3, 127.6, 127.4, 126.3, 125.6, 44.1, 40.7, 35.1, 29.9, 29.8, 26.7, 26.6, 25.4, 24.5. HRMS (ESI) m/z: [M+H]^+^ calcd for C_13_H_17_N_2_O_3_: 249.1234; found: 249.1234. |
| **6.26** *N*-methyl-*N*-(3-methyl-2-(3-oxobutyl)phenyl)nitrous amide **(4m)** | |
|  | The title compound was obtained as pale yellow liquid inseparable mixture of *syn* and *anti* in approximately of 1: 0.14 ratio in 71% yield (78 mg). The residue was purified by column chromatography in silica gel eluting with hexane: EtOAc (80:20), *R_f_* = 0.34; IR (neat) 3409, 3201, 2825, 1772, 1656, 1412, 981, 877, 545. ^1^H NMR (600 MHz, CDCl_3_) δ 7.18 (d, *J* = 7.9 Hz, 1H × 1), 7.13 (d, *J* = 7.9 Hz, 1H × 1), 7.10-7.07 (m, 1H × 0.24), 6.96 (s, 1H × 1), 6.65 (s, 1H × 0.11), 4.00 (s, 3H × 0.44), 3.33 (s, 3H × 1), 2.66 (t, *J* = 7.3 Hz, 2H × 1), 2.61 (t, *J* = 7.3 Hz, 2H × 1), 2.29 (s, 3H × 1), 2.24 (s, 3H × 0.40), 2.02 (s, 3H × 0.40), 2.01 (s, 3H × 1). ^13^C NMR (150 MHz, CDCl_3_) δ 207.4, 207.2, 145.8, 140.8, 137.3, 135.8, 133.9, 130.3, 130.1, 129.4, 126.3, 126.0, 44.4, 43.2, 41.1, 35.4, 34.9, 29.8, 24.8, 24.2, 20.6, 17.8. HRMS (ESI) m/z: [M+H]^+^ calcd for C_12_H_17_N_2_O_2_: 221.1285; found: 221.1282. |
| **6.27** *N*-(3-fluoro-2-(3-oxobutyl)phenyl)-*N*-methylnitrous amide **(4n)** | |
|  | The title compound was obtained as pale yellow liquid inseparable mixture of *syn* and *anti* in approximately of 1: 0.13 ratio in 70% yield (78 mg). The residue was purified by column chromatography in silica gel eluting with hexane: EtOAc (80:20), *R_f_* = 0.33; IR (neat) 3362, 3204, 2829, 1765, 1689, 1342, 987, 845, 546. ^1^H NMR (600 MHz, CDCl_3_) δ 7.27 (q, *J* = 7.3 Hz, 1H × 1), 7.22 (d, *J* = 10.3 Hz, 1H × 0.19), 7.08 (t, *J* = 8.9 Hz, 1H × 1), 7.03 (t, *J* = 8.9 Hz, 1H × 0.17), 6.98 (d, *J* = 7.9 Hz, 1H × 1), 6.68 (d, *J* = 7.9 Hz, 1H × 0.13), 4.03 (s, 1H × 0.40), 3.34 (s, 3H × 1), 2.73 -2.69 (m, 2H × 1), 2.67-2.63 (m, 2H × 1), 2.04 (s, 3H × 1). ^13^C NMR (150 MHz, CDCl_3_) δ 207.2, 206.8, 161.7 (d, *J*_C-F_ = 246 Hz), 142.5 (d, *J*_C-F_ = 10.5 Hz), 139.8 (d, *J*_C-F_ = 4.5 Hz), 128.5 (d, *J*_C-F_ = 3 Hz), 128.4 (d, *J*_C-F_ = 3 Hz), 128.0 (d, *J*_C-F_ = 3 Hz) 127.9 (d, *J*_C-F_ = 3 Hz ), 125.4 *J*_C-F_ = 3 Hz) 125.3 (d, *J*_C-F_ = 3 Hz) 121.4 (d, *J*_C-F_ = 4.5 Hz), 121.1 (d, *J*_C-F_ = 6 Hz) 116.6 (d, *J*_C-F_ = 3 Hz), 115.8 (d, *J*_C-F_ = 3 Hz ), 43.1, 41.7, 41.1, 35.4, 29.6, 29.5, 19.6, 19.3. HRMS (ESI) m/z: [M+H]^+^ calcd for C_11_H_14_FN_2_O_2_: 225.1034; found: 225.1032. |
| **6.28** *N*-methyl-*N*-(2-methyl-6-(3-oxobutyl)phenyl)nitrous amide **(4o)** | |
|  | The title compound was obtained as pale yellow liquid inseparable mixture of *syn* and *anti* in approximately of 1: 0.11 ratio in 72% yield (76 mg). The residue was purified by column chromatography in silica gel eluting with hexane: EtOAc (80:20), *R_f_* = 0.33; IR (neat) 3405, 3241, 2789, 1729, 1689, 1413, 1021, 952, 571. ^1^H NMR (600 MHz, CDCl_3_) δ 7.30 (t, *J* = 7.6 Hz, 1H × 1), 7.23 (d, *J* = 7.7 Hz, 1H × 0.33), 7.21-7.17 (m, 2H × 1), 7.13 (d, *J* = 7.5 Hz, 1H × 0.37), 7.09 (d, *J* = 7.7 Hz, 1H × 0.39), 4.01 (s, 3H × 1), 3.35 (s, 3H × 1), 2.78 (m, 4H × 1.30), 2.67-2.64 (m, 2H × 1), 2.60-2.51 (m, 2H × 1), 2.13 (s, 3H × 1), 2.08 (s, 3H × 1), 2.08 (s, 3H × 1), 2.00 (s, 3H × 1). ^13^C NMR (150 MHz, CDCl_3_) δ 207.0, 140.1, 138.7, 137.8, 135.9, 134.8, 129.7, 129.7 (2C, overlapped), 129.2, 129.0, 127.7, 127.3, 44.4, 43.7, 40.0, 35.1, 29.9 (2C, overlapped), 25.3, 24.9, 17.9, 17.6. HRMS (ESI) m/z: [M+H]^+^ calcd for C_12_H_17_N_2_O_2_: 221.1285; found: 221.1282. |
| **6.29** *N*-ethyl-*N*-(2-methyl-6-(3-oxobutyl)phenyl)nitrous amide (**4r**) | |
|  | The title compound was obtained as pale yellow liquid inseparable mixture of *syn* and *anti* in approximately of 1:1 ratio in 72% yield (84 mg). The residue was purified by column chromatography in silica gel eluting with hexane: EtOAc (75:25), *R_f_* = 0.25; IR (neat) 3357, 3210, 2811, 1824, 1752, 1329, 964, 831, 556. ^1^H NMR (600 MHz,CDCl_3_) δ 7.24 (t, *J* = 7.6 Hz, 1H × 1), 7.15 (m, 3H × 1), 7.07 (d, *J* = 7.5 Hz, 1H × 1), 7.03 (d, *J* = 7.6 Hz, 1H × 1), 4.34 (m, 2H × 1), 3.89 (m,, 1H × 1), 3.78 (m, 1H × 1), 2.72 (m, 1H × 1), 2.61 (m, 3H × 1), 2.50 (m, 3H × 1), 2.39 (m, 1H × 1), 2.09 (s, 3H × 1), 2.02 (s, 6H × 1), 1.95 (s, 3H × 1), 1.40 (t, *J* = 7.3 Hz, 3H × 1), 1.02 (t, *J* = 7.3 Hz, 3H × 1). ^13^C NMR (150 MHz, CDCl_3_) δ 207.4, 207.1, 139.1, 138.5, 138.1, 136.6, 136.3, 135.3, 129.6, 129.5, 129.2, 128.9, 127.6, 127.2, 49.0, 44.5, 43.9, 42.6, 29.8 (2C, overlapped), 25.5, 25.1, 18.2, 18.1, 13.8, 10.7. HRMS (ESI) m/z: [M+H]^+^ calcd for C_13_H_19_N_2_O_2_: 235.1441; found: 235.1440. |
| **6.30** *N*-methyl-*N*-(3-(3-oxobutyl)-[1,1'-biphenyl]-2-yl)nitrous amide (**4p**) | |
|  | The title compound was obtained as pale yellow liquid inseparable mixture of *syn* and *anti* in approximately of 1: 0.03 ratio in 75% yield (105 mg). The residue was purified by column chromatography in silica gel eluting with hexane: EtOAc (75:25), *R_f_* = 0.28; IR (neat) 3415, 3215, 2841, 1780, 1645, 1375, 1011, 892, 543. ^1^H NMR (600 MHz, CDCl_3_) δ 7.40 (t, *J* = 7.6 Hz, 1H × 1), 7.32 (d, *J* = 7.7 Hz, 1H × 1), 7.29-7.24 (m, 4H × 1), 7.12 (d, *J* = 6.4 Hz, 2H × 1), 3.49 (s, 1H × 0.10), 2.80 (s, 1H × 1), 2.79 (s, 3H × 1), 2.68 (t, *J* = 13.1 Hz, 2H × 1), 2.53 (s, 1H × 1), 2.07 (s, 3H × 0.10), 2.04 (s, 3H × 1). ^13^C NMR (150 MHz, CDCl_3_) δ 207.1, 139.7, 139.1, 139.0, 138.2, 129.6, 129.5, 129.1, 128.8, 128.4, 127.6, 44.7, 35.5, 29.8, 25.5. HRMS (ESI) m/z: [M+H]^+^ calcd for C_17_H_19_N_2_O_2_: 283.1441; found: 283.1439. |
| **6.31** *N*-(2-benzoyl-6-(3-oxobutyl)phenyl)-*N*-methylnitrous amide (**4q**) | |
|  | The title compound was obtained as pale yellow liquid inseparable mixture of *syn* and *anti* in approximately of 1: 0.17 ratio in 74% yield (114 mg). The residue was purified by column chromatography in silica gel eluting with hexane: EtOAc (75:25), *R_f_* = 0.32; IR (neat) 3409, 3310, 2815, 1802, 1765, 1359, 972, 852, 539. ^1^H NMR (600 MHz, CDCl_3_) δ 7.72 (dd, *J* = 8.4, 1.3 Hz, 2H × 1), 7.56 -7.53 (m, 2H × 1), 7.50 (s, 2H × 0.29), 7.47 (s, 2H × 0.23), 7.45 (dd, *J* = 7.8, 1.3 Hz, 2H × 0.24), 7.42-7.38 (m, 4H × 1), 7.27 (d, *J* = 1.4 Hz, 2H × 0.45), 4.10 (s, 3H × 0.50), 3.25 (s, 3H × 1), 2.81-2.75 (m, 2H × 1), 2.70 (t, *J* = 7.5 Hz, 2H × 1), 2.09 (s, 3H × 1). ^13^C NMR (150 MHz, CDCl_3_) δ 206.6, 195.5, 139.3, 139.2, 139.1, 138.1, 136.6, 133.6, 133.5, 132.5, 132.1, 130.2, 130.0, 129.1, 128.9, 128.4, 128.3, 127.6, 127.7, 43.9, 43.3, 41.1, 37.0, 36.1, 29.8, 25.0, 24.6. HRMS (ESI) m/z: [M+H]^+^ calcd for C_18_H_19_N_2_O_3_: 311.1390; found: 311.1383. |
| **6.32** *N*-methyl-*N*-(2-(3-oxo-3-phenylpropyl)phenyl)nitrous amide **(4s)** | |
|  | The title compound was obtained as pale yellow liquid inseparable mixture of *syn* and *anti* in approximately of 1:0.14 ratio in 32% yield (43 mg). The residue was purified by column chromatography in silica gel eluting with hexane: EtOAc (75:25), *R_f_* = 0.25; IR (neat) 3356, 3158, 2845, 1856, 1755, 1362, 989, 874, 552. ^1^H NMR (600 MHz,CDCl_3_) δ 7.82 (d, *J* = 7.2 Hz, 2H × 1), 7.47 (d, *J* = 7.4 Hz, 1H × 1), 7.38-7.34 (m, 4H × 1), 7.30 (d, *J* = 2.0 Hz, 1H × 0.48), 7.26 (m, 1H × 0.46), 7.22 (d, *J* = 3.1 Hz, 1H × 0.57), 7.21-7.19 (m, 1H × 1), 7.16 (d, *J* = 8.8 Hz, 1H × 1), 4.04 (s, 3H × 0.42), 3.36 (s, 3H × 1), 3.17 (t, *J* = 7.2, 2H × 1), 2.90 (t, *J* = 7.6 Hz, 2H × 1). ^13^C NMR (150 MHz, CDCl_3_) δ 198.6, 141.2, 140.8, 136.4, 133.1, 130.7, 129.9, 129.4, 128.5, 127.9, 127.5, 126.9, 125.9, 125.2, 41.2, 39.7, 38.5, 35.5, 25.8, 25.1. HRMS (ESI) m/z: [M+H]^+^ calcd for C_16_H_16_N_2_O_2_: 269.1285; found: 269.1282. |
| **6.33** *N*-methyl-*N*-(4-methyl-2-(3-oxo-3-phenylpropyl)phenyl)nitrous amide **(4t)** | |
|  | The title compound was obtained as pale yellow liquid inseparable mixture of *syn* and *anti* in approximately of 1:0.14 ratio in 17% yield (24 mg). The residue was purified by column chromatography in silica gel eluting with hexane: EtOAc (75:25), *R_f_* = 0.25; IR (neat) 3319, 3215, 2833, 1862, 1759, 1359, 965, 889, 565. ^1^H NMR (600 MHz, CDCl_3_) δ 7.83 (d, *J* = 7.9 Hz, 2H × 1), 7.48 (d, *J* = 7.3 Hz, 1H × 1), 7.36 (t, *J* = 7.8 Hz, 3H × 1), 7.28 (s, 1H × 0.45), 7.18 (d, *J* = 10.1 Hz, 2H × 1), 7.08 (s, 1H × 0.49), 7.05 (s, 1H × 0.44), 4.02 (s, 1H × 0.60), 3.35 (s, 3H × 1), 3.18-3.15 (m, 2H × 1), 2.87-2.84 (m, 2H × 1), 2.32 (s, 3H × 1). ^13^C NMR (150 MHz, CDCl_3_) δ 198.7, 139.58, 138.8, 137.2, 136.5, 133.1, 131.3, 128.5, 128.0, 125.8, 39.9, 35.6, 25.8, 21.1. HRMS (ESI) m/z: [M+H]^+^ calcd for C_17_H_19_N_2_O_2_: 283.1441; found: 283.1437. |
| **6.34** 4-(1-methyl-2,3-diphenyl-1H-indol-7-yl)butan-2-one **(5a)** | |
|  | The title compound was obtained as pale yellow liquid in 43% yield (76 mg). The residue was purified by column chromatography in silica gel eluting with hexane: EtOAc (88:12), *R_f_* = 0.49; IR (neat) 3317, 3152, 2813, 1878, 1762, 1329, 971, 892, 554. ^1^H NMR (600 MHz, CDCl_3_) δ 7.54 (d, *J* = 8.8 Hz, 1H), 7.31-7.27 (m, 3H), 7.23 (dd, *J* = 7.3, 2.1 Hz, 2H), 7.18 (d, *J* = 3.4 Hz, 4H), 7.10 (m, 1H), 7.01 (t, *J* = 7.5 Hz, 1H), 6.96 (d, *J* = 6.7 Hz, 1H), 3.75 (s, 3H), 3.40-3.35 (m, 2H), 2.89-2.85 (m, 2H), 2.14 (s, 3H). ^13^C NMR (150 MHz, CDCl_3_) δ 207.7, 139.3, 135.7, 135.0, 131.9, 131.3, 130.0, 128.6, 128.3, 128.0, 128.0, 125.5, 124.6, 124.0, 120.4, 118.1, 115.9, 45.8, 34.3, 30.2, 26.5. HRMS (ESI) m/z: [M+H]^+^ calcd for C_25_H_24_NO: 354.1852; found: 354.1851. |
| **6.35** *N*-(2-benzoyl-6-(3-oxobutyl)phenyl)-*N*-methylnitrous amide (**5b**) | |
|  | The title compound was obtained as pale yellow liquid in 32% yield (50 mg). The residue was purified by column chromatography in silica gel eluting with hexane: EtOAc (75:25), *R_f_* = 0.32; IR (neat) 3409, 3312, 2817, 1802, 1762, 1359, 972, 852, 539. ^1^H NMR (600 MHz,CDCl_3_) δ 7.67 (d, *J* = 8.0 Hz, 2H), 7.50 (t, *J* = 7.4 Hz, 2H), 7.44 (t, *J* = 7.6 Hz, 1H), 7.35 (t, *J* = 7.8 Hz, 3H), 3.20 (s, 3H), 2.73 (t, *J* = 6.3 Hz, 2H), 2.65 (t, *J* = 7.3 Hz, 2H), 2.05 (s, 3H). ^13^C NMR (150 MHz, CDCl_3_) δ 206.6, 195.5, 139.4, 139.1, 138.1, 136.6, 133.6, 132.6, 130.1, 129.1, 128.4, 127.7, 43.9, 37.0, 29.9, 25.1. HRMS (ESI) m/z: [M+H]^+^ calcd for C_18_H_19_N_2_O_3_: 311.1390; found: 311.1388. |

1. **References**
2. P. Chaudhary, S. Gupta, N. Muniyappan, S. Sabiah, J. Kandasamy, *Green. Chem.,* **2016**, *18*, 2323**-**2330.
3. B. Q. Liu, C. Song, C. Sun, S. G. Zhou, J. Zhu, *J. Am. Chem. Soc*., **2013**, *135*, 16625**-**16631.
4. Y. Wu, L. Sun, Y. Chen, Q. Zhou, J.-W. Huang, H. Miao, H-B Luo, *J. Org. Chem.,* **2016**, *81*, 1244-1250.
5. **^1^H NMR and ^13^C NMR spectra of synthesized compounds**

__

__

__

__

__

__

__

__

__

__

__

__

__

__

__

__

__

__
